# A rare *LMNA* missense mutation causing a severe phenotype of mandibuloacral dysplasia type A: a case report

**DOI:** 10.1590/1984-0462/2024/42/2022189

**Published:** 2024-05-27

**Authors:** Adriana Amaral Carvalho, Renato Assis Machado, Célia Márcia Fernandes Maia, Luis Antônio Nogueira dos Santos, Daniella Reis Barbosa Martelli, Ricardo Della Coletta, Hercílio Martelli

**Affiliations:** a Universidade Estadual de Montes Claros, Montes Claros, MG, Brazil.; b Universidade Estadual de Campinas, Piracicaba, SP, Brazil.

**Keywords:** Progeria, Acro-osteolysis, Lipodystrophy, Mandibuloacral dysplasia, LMNA, Progeria, Acro-osteólise, Lipodistrofia, Displasia mandibuloacral, LMNA

## Abstract

**Objective:**

To report the case of a girl presenting a severe phenotype of mandibuloacral dysplasia type A (MADA) characterized by prominent osteolytic changes and ectodermal defects, associated with a rare homozygous *LMNA* missense mutation (c.1579C>T).

**Case description:**

A 6-year-old girl was evaluated during hospitalization exhibiting the following dysmorphic signs: subtotal alopecia, dysmorphic facies with prominent eyes, marked micrognathia and retrognathia, small beaked nose, teeth crowding and thin lips, generalized lipodystrophy, narrow and sloping shoulders, generalized joint stiffness and bone reabsorption in the terminal phalanges. In dermatological examination, atrophic skin, loss of cutaneous elasticity, hyperkeratosis, dermal calcinosis, and hyperpigmented and hypochromic patches were observed. Radiology exams performed showed bilateral absence of the mandibular condyles, clavicle resorption with local amorphous bone mass confluence with the scapulae, shoulder joints with subluxation and severe bone dysplasia, hip dysplasia, osteopenia and subcutaneous calcifications.

**Comments:**

MADA is a rare autosomal recessive disease caused by mutations in *LMNA* gene. It is characterized by craniofacial deformities, skeletal anomalies, skin alterations, lipodystrophy in certain regions of the body and premature ageing. Typical MADA is caused by the p.R527H mutation in the *LMNA* gene. However, molecular analysis performed from oral epithelial cells obtained from the patient showed the rare mutation c.1579C>T, p. R527C in the exon 9 of *LMNA.* This is the sixth family identified with this mutation described in the literature.

## RESUMO

**Objetivo:**

Relatar o caso de uma jovem que apresentava um fenótipo grave de displasia mandibuloacral tipo A (MADA) caracterizado por alterações osteolíticas proeminentes e defeitos ectodérmicos, associados a uma rara mutação homozigótica missense no gene *LMNA* (c.1579C>T).

**Descrição do caso:**

Uma menina de seis anos foi avaliada durante hospitalização apresentando os seguintes sinais dismórficos: alopecia subtotal, fácies dismórfica com olhos proeminentes, micrognatia e retrognatia acentuada, nariz pequeno e adunco, dentes apinhados e lábios finos, lipodistrofia generalizada, ombros estreitos e inclinados, rigidez articular e reabsorção óssea nas falanges terminais. Ao exame dermatológico, observou-se pele atrófica, perda da elasticidade cutânea, hiperceratose, calcinose dérmica e manchas hiperpigmentadas e hipocrômicas. Exames radiológicos realizados mostraram ausência de côndilos mandibulares bilaterais, reabsorção da clavícula com massa óssea amorfa local confluindo com as escápulas, articulações do ombro com subluxação e displasia óssea severa, com displasia coxofemoral, osteopenia e calcificações subcutâneas.

**Comentários:**

MADA é uma doença autossômica recessiva rara causada por mutações no gene *LMNA.* Caracteriza-se por deformidades craniofaciais, anomalias esqueléticas, alterações cutâneas, lipodistrofia em determinadas regiões do corpo e envelhecimento precoce. MADA típica é causada pela mutação p.R527H no gene *LMNA.* No entanto, a análise molecular realizada com células epiteliais orais obtidas da paciente mostrou a mutação rara c.1579C>T, p. R527C no exon 9 do gene *LMNA.* Esta é a sexta família identificada com essa mutação descrita na literatura.

## INTRODUCTION

Mandibuloacral dysplasia (MAD) is a genetic condition belonging to the category of laminopathies, that comprise the progeroid syndromes and lipodystrophies, in which MAD is included, as well as muscular dystrophies, cardiomyopathies and hereditary peripheral neuropathies.^1,2^ It is a rare autosomal recessive disorder characterized by craniofacial anomalies, skeletal abnormalities including generalized osteoporosis, progressive osteolysis of the distal phalanges and clavicles, growth retardation, joint contractures, pigmentary and atrophic skin changes, lipodystrophy signs and mildly accelerated ageing.^3,4^

Patients with MAD are categorized according to trends of lipodystrophy in types A and B, which are linked to different genetic defects. Mutations in *LMNA,* which encodes lamin A and C, are associated to type A pattern of lipodystrophy (MADA; OMIM #248370) and mutations on the zinc metalloproteinase *ZMPSTE24,* encoding a protease involved in post-translational proteolytic processing of prelamin A to form mature lamin A, are linked to type B lipodystrophy (MADB; OMIM #608612).^4,5^

This study reports the clinical case of a 6-year-old girl presenting a severe phenotype linked to *LMNA* mutation.

## CASE REPORT

The 6-year-old girl is the eldest of three children belonging to a consanguineous couple. The patient in this report was born at term, weighing 2.9 kg and measuring 50 cm, by vaginal delivery, Apgar score of 8/9. In the neonatal period, she evolved with an ischemic stroke in the left middle cerebral artery with hemorrhagic transformation probably associated to polycythemia (hematocrit=69%). However, it was not possible to define by imaging exams whether the alteration had occurred intrauterinely or postnatally. The child was followed up in a hematology service until the age of four, where tests were carried out for hereditary thrombophilia and vasculitis.

Polymerase chain reaction (PCR) analysis performed at 21 months of age evidenced the presence of the factor V Leiden mutation in heterozygosis, an alteration that possibly contributed to the stroke in the neonatal period. There were no mutations in the prothrombin gene, and low levels of factor VIII were reported (39%). Antibody testing for anticardiolipin and lupus anticoagulant, which may be associated with thrombosis, was negative, as well as the investigation for systemic sclerosis, through the determination of anti-fibrillarin, anticentromere, and anti-DNA topoisomerase I antibodies. Tests to identify antinuclear antibodies (ANA), rheumatoid factor, and C-reactive protein were normal.

Regarding phenotypic changes, the patient at birth had a normal appearance but during the first year of life mild abnormalities appeared, such as hair loss and whitish lesions in the joints of the hands at four months of age, and hyperchromic spots on the body at six months. She evolved with normal developmental parameters, having acquired independent walking at 14 months. At the age of two, she was evaluated in a specialized hospital and, according to the medical report, presented weight, height, and head circumference below the 3^rd^ percentile, dysmorphic face characterized by thin skin, sharp nose, hypoplastic malar region, prominent cranial veins, irregular skin discoloration, shortage of subcutaneous fat, rigid finger joints, and fine hair with alopecia in the occipital region.

Laboratory tests performed, such as a screening for innate metabolism errors and organic acidemias, transferrin isoelectric focusing, molecular genetic test for mitochondrial encephalomyopathy, lactic acidosis, and stroke-like episodes (MELAS), karyotype, serum dosage of thyroxine, thyroid stimulating hormone, aspartate aminotransferase and alanine aminotransferase, were all normal. Imaging exams revealed osteolysis of the distal phalanx (fingers and toes), decreased bone density, deformity of the hip joints, hypoplasia of the head of the radius and clavicles, widening of the sagittal sutures.

**Figure 1:**
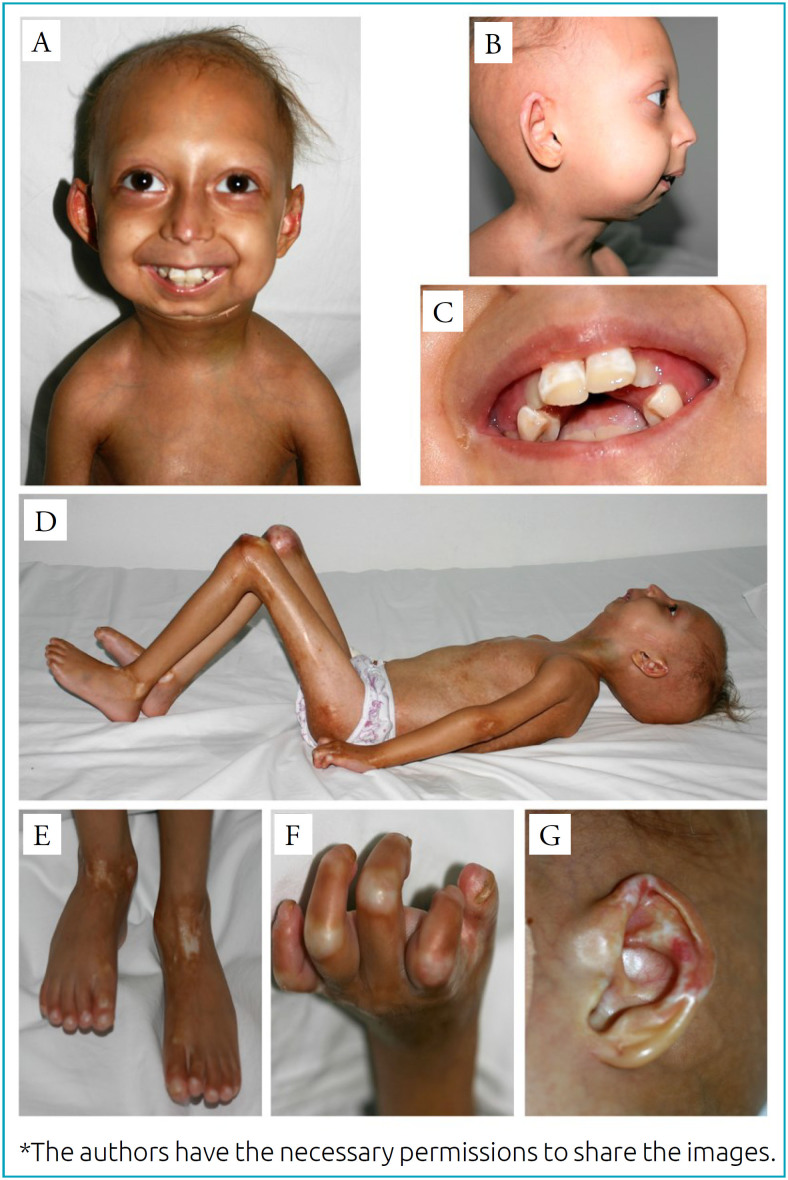
Patient pictures (A: frontal and B: lateral view of the patient, with dysmorphic signs: subtotal alopecia, prominent eyes, marked micrognathia and retrognathia, small beaked nose; C: image show thin lips and teeth crowding; D: full body view of the patient, with generalized joint stiffness with abnormal posture and decreased mobility; E, F, G: dermatological changes of the patient, with hypochromic patches, bone reabsorption in the terminal phalanges and dysplastic nails).

The patient displayed progressive clinical worsening. At four years of age, after a trauma in both knees, the joints were fixed in a flexible position, and she was unable to walk anymore. At six year of age, the child had the following dysmorphic signs: subtotal alopecia, dysmorphic facies with prominent eyes, marked micrognathia and retrognathia, small beaked nose, teeth crowding and thin lips (Figures 1A, 1B and 1C), generalized lipodystrophy, narrow and sloping shoulders, generalized joint stiffness (Figure 1D), and bone reabsorption in the terminal phalanges (Figures 1E and 1F).

**Figure 2:**
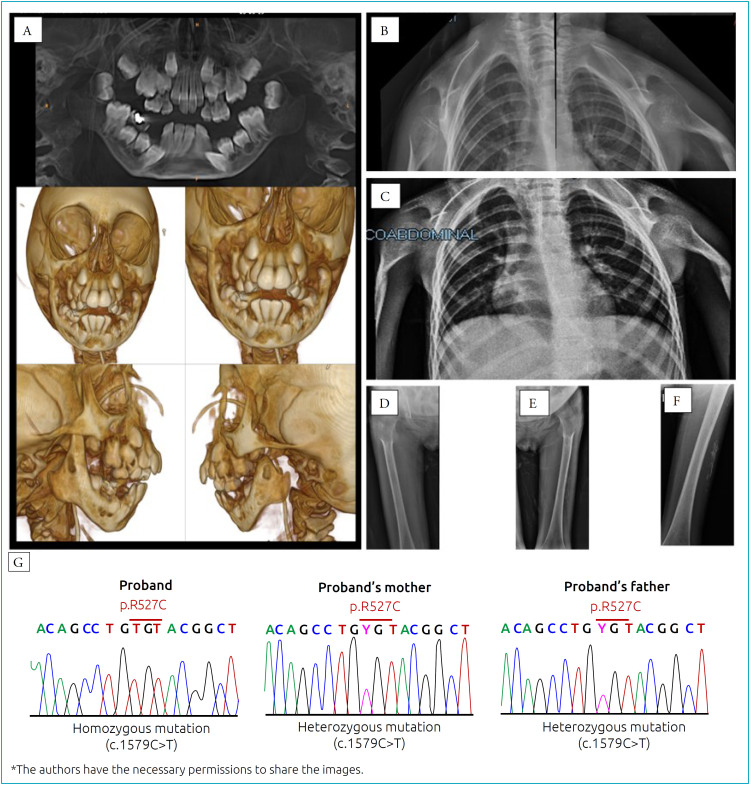
Exams result (A: cone-beam computed tomography images: hypoplasia of the mandible branch, with bilateral absence of the mandibular condyles and teeth crowding; B: radiographic findings: clavicle absent, with the presence of local amorphous bone mass confluent with the scapulae; C: humerus and shoulder joints with subluxation and major bone dysplasia; D: high hip dysplasia; E: left hip dysplasia; F: significant osteopenia and subcutaneous calcification; G: sequencing of the patient's genetic material and her parents).

In dermatological examination, atrophic skin, loss of cutaneous elasticity, hyperkeratosis, dermal calcinosis, and hyperpigmented and hypochromic patches were observed (Figures1D to 1G). Diffuse gingivitis with bleeding and presence of significant crowding of the teeth were noted. The marked microretrognathia and glossoptosis, causing significant upper airway obstruction, culminated in the placement of a tracheostomy. The computed tomography (CT) scan of the facial bones allowed the detection of hypoplasia of the mandible branch, with absence of the condyles bilaterally and teeth crowding (Figure 2A).

Further radiographic exams revealed clavicles bilaterally absent with the presence of local amorphous bone mass confluent with the scapulae (fused acromioclavicular joints) (Figure 2B), humerus and sho ulder joints with subluxation and major bone dysplasia (Figure 2C), hip dysplasia (Figures 2D and 2E), significant osteopenia, and subcutaneous calcifications (Figures 2E and 2F). Cardiac CT performed to assess the coronary calcium score did not demonstrate coronary artery disease.

Complete hemogram, renal and liver function tests, serum glucose, parathormone levels, calcium, phosphorus, alkaline phosphatase and 25-hydroxy vitamin D were within normal limits. High levels of cholesterol (191 mg/dL, normal range: <150 mg/dL) were detected, and serum lipid profiles showed an increase in low-density lipoprotein cholest erol (102 mg/dL, normal range: <100 mg/dL).

The genomic DNA isolated from oral epithelial cells was amplified by PCR with primers designed to all exons and flanking intronic regions of *LMNA* and *ZMPSTE24* described (http://www.hgmd.cf.ac.uk/ac/all.php, Supplementary Table 1). After amplification, PCR products were cleaned up and sequenced with a BigDye Terminator v3.1 Cycle Sequencing kit (Applied Biosystems, CA, USA). The sequences were analyzed with a 3500 Genetic Analyzer (Applied Biosystems, CA, USA). The genetic analysis detected the mutation c.1579C>T, p. R527C in the exon 9 of *LMNA*, compatible with the diagnosis of MADA. The analysis of the patient's parents confirmed the autosomal recessive transmission of the c.1579C> T mutation in exon 9 of the *LMNA* gene (Figure 2G).

## DISCUSSION

Progeroid laminopathies are a group of rare genetic disorders characterized by the premature appearance of certain signs of physiological aging in a subset of tissues.^6^ Within the group, Hutchinson-Gilford progeria syndrome (HGPS) is the most common type and this was the diagnosis initially established for the patient. It represents the most widely studied condition in this group of disorders.^7,8^ Interestingly, while bone, skin, and adipose tissue are severely affected in all forms of *LMNA*-linked progeria, the cardiovascular system is selectively involved in the HGPS. Atherosclerosis is the most important clinical manifestation, considering that this is the cause of premature death in these patients from myocardial infarction or stroke in early adolescence.^8,9^ Our patient was submitted to a cardiac CT to assess the coronary calcium score, without evidence of coronary artery disease.

In addition to HGPS, the progeroid laminopathies include MADA, MADB, Nestor-Guillermo progeria syndrome (NGPS)^7^ and restrictive dermopathy. While HGPS and MADA are linked to mutations in the *LMNA* gene, MADB and restrictive dermopathy are associated with a mutation in *ZMPSTE24*, a gene that encodes a zinc metalloproteinase necessary for the correct processing of prelamin A to mature lamin A.^5,10^ NGPS, on the other hand, is due to a mutation in the *BANF1* gene, which plays key roles in gene expression, nuclear assembly and chromatin organization.^11^ The genetic analysis performed in the patient revealed the mutation c.1579C>T (p.R527C) in exon 9 of the *LMNA* gene, leading to the diagnosis of MADA.

Pronounced acro-osteolysis is a signature symptom of MAD.^10,12^ Typical MADA is caused by the p.R527H mutation in the *LMNA* gene, while other *LMNA* mutations are responsible for different phenotypes.^8^ Regarding the phenotype, patients with MADA usually have distinguished features compared to MADB, which include more severity of clinical phenotype, early onset, renal disease, calcified skin nodules, premature birth and lack of acanthosis nigricans in the MADB type.^4,10,12^ Thus, a milder phenotype and slower disease progression is generally expected in MADA.

However, the patient described in this case report had an earlier onset of symptoms and more severe phenotype than that usually described for MADA. In agreement with these findings, patients already identified with this mutation have been described in the literature as presenting a similar phenotype and clinical course. Agarwal was the first to describe this mutation, in 2008.^12^ The clinical manifestations highlighted by him that denote the precocity of onset and severity of the phenotype associated are growth impairment already apparent at one year of age; joint stiffness, skin thinning, and mottled hyperpigmentation developed by 15 months of age; bird face with beak nose, bulbous forehead and very small mouth with limited opening capacity seen at two years of age; progressive clavicular hypoplasia, acro-osteolysis and severe hair loss in the temporal and occipital areas described at three years of age; difficulty swallowing and severe snoring with obstructive apnea; very crowded teeth; lipodystrophy evidenced in the first years of life; severe joint contractures, resulting in abnormal posture and decreased mobility.^12^

All these alterations described by Agarwal were also observed at an early age in the patient in this report.^12^ In addition, radiology exams performed at six years of age showed absence of bilateral mandibular condyles, clavicle resorption with local amorphous bone mass confluence with the scapulae, humeral and shoulder joints with subluxation and severe bone dysplasia, hip dysplasia, osteopenia and subcutaneous calcifications, the latter a feature commonly described in MADB. Another important consideration is that biochemical tests performed revealed metabolic disorder with the presence of hypercholesterolemia and increased low-density lipoprotein cholesterol, probably associated with the generalized lipodystrophy observed in our patient, a pattern of lipodystrophy typically expected for MADB.^4,5,13^

Liang in 2009 also reported the case of two Chinese brothers who presented the homozygous c.1579C>T (R527C) mutation found in our patient, which he describes as having atypical HGPS, not MADA.^14^ The presence of electrocardiogram (ECG) abnormalities suggesting myocardial ischemia evidenced in the older patient is a commonly expected finding in patients with HGPS. However, the intense osteolysis present at an early age in both siblings is a sign of MAD. In addition, the presence of full cheeks was also a finding contrary to what is expected for typical HGPS. Based on the analysis of the clinical data and the result of the molecular analysis performed by Liang, the evidence points, in our opinion, that the probable diagnosis of the two children was MADA, and not atypical HGPS as described by the authors.

In another study carried out, patients descended from three unrelated consanguineous families were evaluated.^15,16^ The same c.1579 C>T – p.(Arg527Cys) mutation identified in our study was found in two of the evaluated families. The age at onset of symptoms in the patients was considerably younger than usually described for MADA. These findings are consistent with the results of this research, which is the association of the mutation c.1579C>T with severe MADA disorder.

The same mutation was found by Luo in 2014, in three Chinese sisters.^4^ He also reports that the phenotype of the patients was more severe than those previously reported in MADA. As a manifestation not previously described for patients with this mutation, there was the possibility of associated muscle damage in two of the sisters, based on the increase in phosphocreatine and an altered electromyography result observed.

Petillo, in 2015, reported the clinical case of four individuals who had a *de novo* mutation in the *LMNA* gene. Among these, the genetic study of patient 4 identified the mutation c.1579C>T described as being a *de novo* missense pathogenic mutation in autosomal dominant inheritance.^17^ The genetic analysis carried out in our research detected the same mutation c.1579C>T, but with recessive transmission, confirmed by analysis of DNA sample obtained from parents. It is know that any direct correlation between mutations in the *LMNA* gene and clinical manifestations is hampered by the pleiotropic effect possibly exerted by Lamin A/C gene mutations.^18^ These pieces of evidence probably explain the very different clinical manifestations in this case when compared to the phenotype observed in our patient, as well as in other cases in the literature reported in this study, and the divergent diagnosis established as Emery-Dreifuss muscular dystrophy.

Of great relevance for the specific diagnosis of genetic syndromes are the recent advances obtained with the use of machine-learning algorithms for image analysis in clinical practice. Through the use of artificial intelligence in the evaluation of images of patients, it is possible to detect similarities of new phenotypes with known disorders and, thus, identify a potential molecular etiology. Using two different image analysis algorithms as a tool, Marbach et al. correctly identified that two individuals from different ethnic backgrounds had highly similar facial dysmorphology and phenotypic proximity to other nuclear envelopathies.^19^ The hitherto unknown genetic disorder in both was identified as being caused by the same *de novo* mutation in *LEMD2*. This disorder exhibits a distinct combination of symptoms with phenotypic overlap with other established segmental progeroid syndromes described in the present study, but with an overall better prognosis when compared to these related syndromes.^19^

In conclusion, this study led to the identification of an unusual mutation in the *LMNA* gene, which contributes to the pathogenicity of MADA. This is the sixth family identified with this mutation described in the literature. The clinical manifestations reported so far in patients with the c.1579C>T mutation are consistent with a more severe phenotype, with an earlier age of onset of osteolysis, alopecia, and lipodystrophy. Given the importance and multiple functions of lamina A and lamina C in maintaining the structure and function of the cell nucleus, it would be expected that mutations in the *LMNA* gene could cause different types of genetic disorders, with different phenotypes. Thus, mutations in different locations of this gene can cause different laminopathies, and even mutations in the same location, but with different amino acid substitutions, can induce great clinical heterogeneity.
